# Effects of irrigation systems and water management strategies on soil chemical properties and citrus tree productivity in clayey soils

**DOI:** 10.1038/s41598-025-19575-6

**Published:** 2025-09-29

**Authors:** Abousrie A. Farag, Mohamed A. Abd El-Aziz, Ahmed. M. El-Husseiny

**Affiliations:** 1 Department of Agricultural and Biosystems Engineering, Faculty of Agriculture, Benha University, Banha, Egypt; 2 Agricultural Research Center (ARC), Soil, Water and Environment Research Institute (SWERI), Giza, Egypt

**Keywords:** Surface irrigation, Subsurface drip irrigation, Deficit irrigation, Soil salinity, Nutrient uptake, Crop productivity, Chemistry, Engineering, Salt

## Abstract

The growing demand for irrigated crops, coupled with water scarcity and climate change, has made the adoption of efficient irrigation systems and water-saving strategies essential. However, concerns remain regarding the long-term effects of localized soil wetting and deficit irrigation (DI) on soil health and crop productivity, particularly in clayey soils. In that concern, a three-season study was conducted on citrus trees in clayey soils at the Faculty of Agriculture farm, Benha University, Egypt, to evaluate the application effects of surface (FDI) and subsurface drip irrigation (SDI) systems, with and without deficit irrigation, on soil properties and crop yield, compared to traditional flood irrigation (FI). Treatments was conducted as Deficit surface drip irrigation (DFDI), Deficit sub-surface drip irrigation (DSDI), full surface drip irrigation (FFDI) and full sub-surface drip irrigation (FSDI). Under full water requirements (FWR), FSDI outperformed FFDI and FI in terms of water savings (31.58%), water use efficiency (WUE) (58.87%), nutrient uptake (N-P-K) (2.44, 10.52, and 5.69%, respectively), and yield (8.70%), with the lowest rates of deterioration over time. In contrast flood irrigation, despite its higher water consumption, it maintained lower levels of root-zone salinity, alkalinity, and sodicity. Under deficit irrigation (DI), DSDI achieved the highest water savings (48.68%), followed by DFDI at 45.82%. However, applying DI caused the highest deterioration rates over time under both irrigation systems for all studied parameters.

## Introduction

The growing global population demands increased agricultural production, often limited by water scarcity and soil salinization, particularly in arid regions^1^. In Egypt, agriculture consumes more than 85% of available water, worsened by climate change, necessitating effective irrigation techniques^2,3^. Water-saving methods like deficit irrigation enhance productive sustainability^4^. Also, replacing flood irrigation with efficient drip irrigation enhances productive sustainability^5,6^. Drip irrigation targets the root zone that shrinks the moistened soil area by about 30% compared to other surface irrigation systems, increases and regulates water content, reduces water use up to 50%, and increases yields^7,8^. It also optimizes water efficiency^9,10^.

On the other hand, Arid and semi-arid Egyptian soils experience excessive salt buildup in the topsoil, negatively impacting crop growth and yield^11,12^, This issue is particularly problematic in the Nile Valley Delta, where loamy to clayey soils dominate^13^.

These soils have poor hydraulic properties, limited drainage, and a high capacity to retain exchangeable cations, all contributing to the salinization problem. Furthermore, practices such as seawater intrusion, poor irrigation techniques, and excessive fertilizer use exacerbate these issues^3,11^. Over the past two decades, drip irrigation has been studied for its potential to manage salt-affected soils by pushing salts to the edges of the wetting zone. Under optimal conditions, short-term use of drip irrigation (less than 5 years) has been found to reduce root-zone salinity and improve crop yield by flushing salts into deeper soil layers, all while using less water^14,15^. However, under suboptimal conditions and with long-term use (over 5 years), drip irrigation can lead to secondary salinization in 60–80% of cases^16–18^.

Unlike flood irrigation, drip irrigation produces an ellipsoidal wetting pattern^8,10^. Soil texture significantly influences the wetting pattern shape due to its impact on hydraulic conductivity and water retention^19^. In contrast to sandy soils, the pattern at clayey soils is wider horizontally than depth^20,21^. Crop roots often concentrate in shallow salt-leaching zones, further threatening productivity^22^.

Drip irrigation systems, with low discharges and point-source water delivery, disrupts water and salinity distribution in the root zone, especially in limited leaching potential soils, where the balance between salt leaching and its accumulation is affected by infiltration and evaporation dynamics of irrigation water^17,23,24^, where salts being leached from the wetted area and accumulating at the wetting front, creating a desalinated zone under the drip tape^25^. After irrigation, evapotranspiration draws salts upward from deeper soil layers, complicating salt removal and leading to further salinization^26^, which contributes to soil degradation and significant yield losses^15^.

Deficit irrigation involves applying less water than required to improve water-use efficiency, which can further promote salt accumulation and reduce yields^27^. While deficit irrigation is beneficial in water-scarce areas by conserving water and improving certain crop qualities^4^, it carries the risk of harming water-sensitive plants and long-term soil health due to inadequate salt flushing^27,28^.

In Egypt, the application of water conservation strategies in clayey soils is often hindered by soil and productivity deterioration over time. Previous studies have focused primarily on reducing water use and improving yield, ignoring the mutual impacts of soil properties on efficiency and sustainability. To address this gap, a three-season study was conducted to investigate the effects of drip irrigation (surface and subsurface) with and without deficit irrigation on water consumption, soil properties, and crop yield, in comparison with flood irrigation systems.

## Materials and methods

### Study location and experimental design

**Fig. 1:**
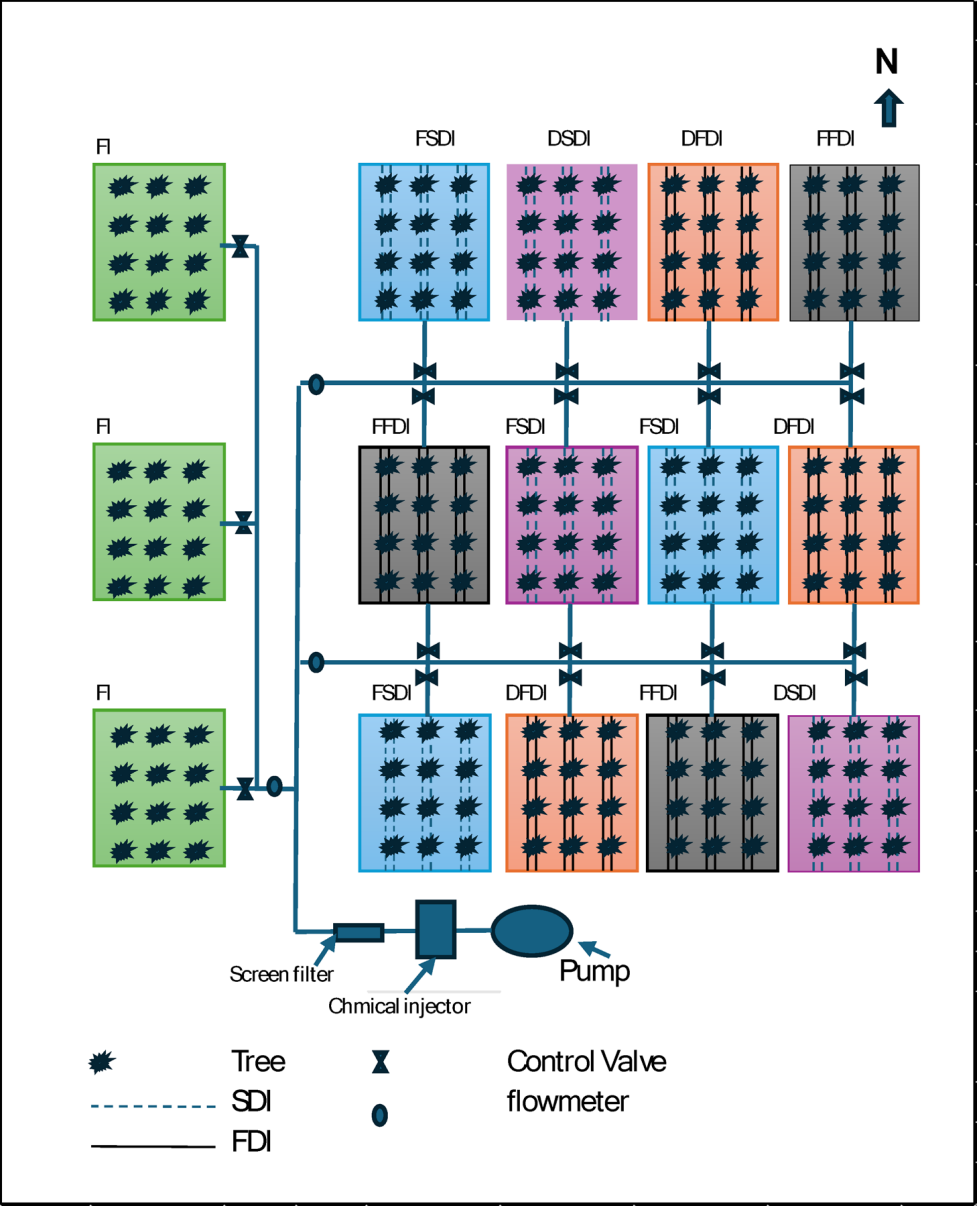
Layout of the field experimental design showing the distribution of irrigation treatments for citrus trees. FI = flood irrigation; FDI = fixed drip irrigation; SDI = subsurface drip irrigation; FSDI = full subsurface drip irrigation; DSDI = deficit subsurface drip irrigation; DFDI = deficit fixed drip irrigation; FFDI = full fixed drip irrigation.

**Fig. 2:**
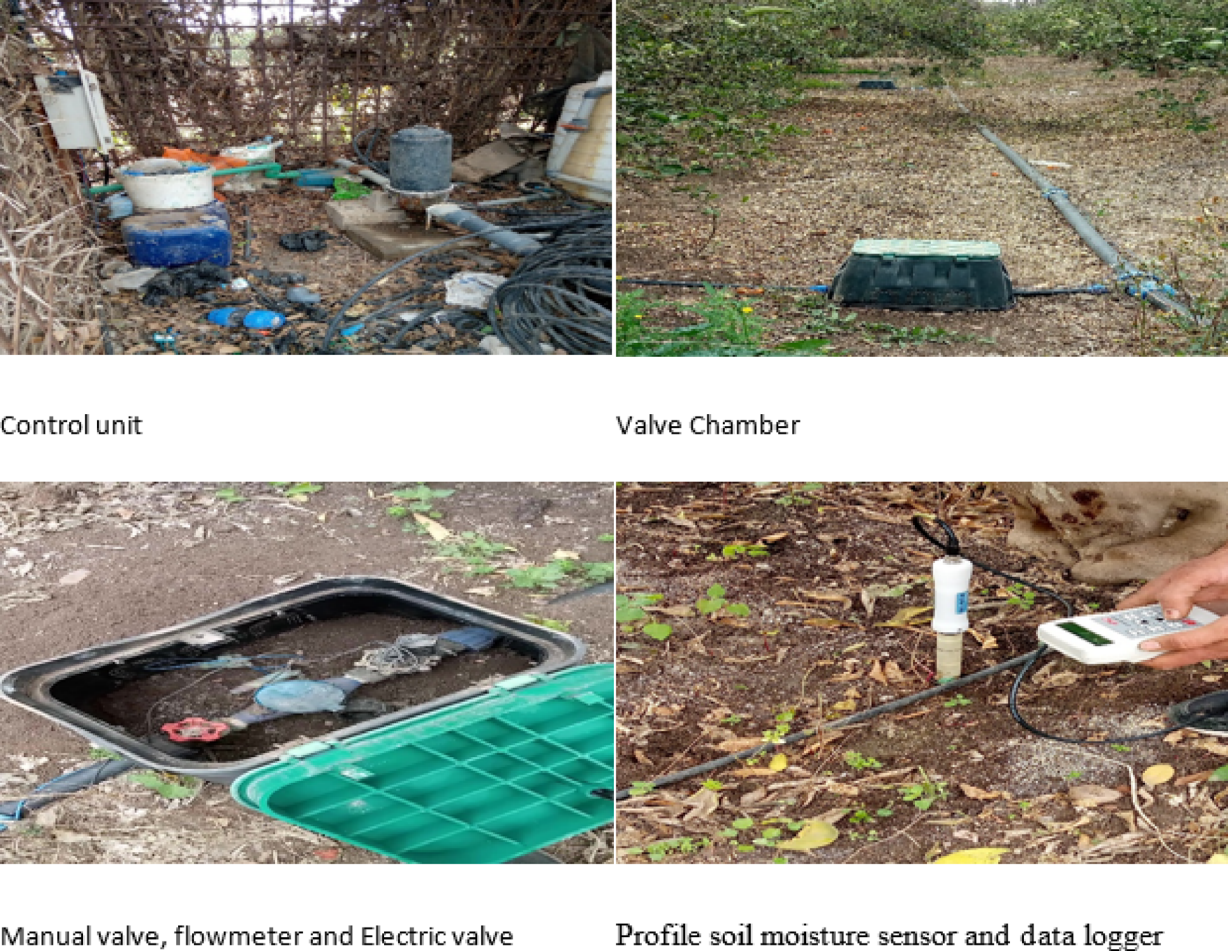
Irrigation systems components and soil water content measurement devices.

This study was conducted in a 20-year-old Navel orange (Citrus sinensis L.) orchard at the Citrus Tree Farm, Benha University, Egypt (30°21’26.9"N, 31°13’22.4"E), over three seasons from 2021 to 2024. A strip plot design field experiment compared surface (FDI) and subsurface drip irrigation (SDI) systems with two irrigation strategies, full water requirements (FWR), and deficit irrigation (DI). flood irrigation (FI) was the control treatment. Each treatment was triple replicated, by 12 trees, totaling 180 citrus trees (Figs. 1 and 2).

### Irrigation system setup

The irrigation was supplied using groundwater via a drip irrigation system powered by a 3 hp pump. The system included a screen filter, a 3-bar manometer, a regulator, and 75 , 63 and 32 mm PVC pipes (main sub-main and manifolds), with control valves, analog water meters, and  16 mm lateral pipes encircling each tree. The system provided irrigation at the soil surface for FDI and at a 30 cm depth for SDI, with four in-line crosslinked emitters with a flow rate of 4 L.hr^− 1^ each.

Meteorological data were collected from the local meteorological station (Embiant110 with WiFi, Ambient, USA) for estimating reference evapotranspiration (ETo). Irrigation volumes were calculated following the method of^29^, with crop evapotranspiration (ETc) estimated using the equation:

1 $${\text{ETc (mm/day)}} = {\text{ETo}} \times {\text{Kc}}$$

Where ETc is the crop evapotranspiration, ETo is the reference evapotranspiration, and Kc is the crop coefficient, as referenced from^30^.

The total available water (TAW) was calculated as:

2 $$TAW (mm) = \left( {\theta _{{FC}} - \theta _{{WP}} } \right) \times {\text{rooting depth (mm)}}$$

Where *θ*_*FC*_ (cm^3^ cm^-3^) is the field capacity and *θ*_*WP*_ (cm^3^ cm^-3^) is the permanent wilting point.

Readily available water was estimated by:

3 $$RAW (mm)= p \times TAW$$

Where *p* is the soil water depletion factor, and TAW is the total available water.

Irrigation requirements (IR) were estimated by:

4 $$IR = ETc - P_{{effective}}$$

Where IR is Irrigation requirements, ETc is the crop evapotranspiration, and P is the soil water depletion factor.

Irrigation requirements (IR) were detailed in (Table 1), and tracked seasonally using counters for FDI and pump discharge timing for FI. DI was applied by reducing 25% of crop evapotranspiration (ETc). Applying the deficit irrigation (DI) by reducing 25% of crop evapotranspiration (ETc) was based on previous studies and recommendations that demonstrate the feasibility of moderate deficit levels in citrus production. Applying 75% ETc means that irrigation water applied under the DI treatment was reduced by 25% compared to full irrigation, aiming to optimize water use while minimizing negative impacts on yield and fruit quality.

The 25% reduction was chosen as a moderate DI level, which is commonly tested in citrus research to balance water savings and acceptable agronomic performance. Several studies (e.g^31–33^. , have reported that irrigation at 75–80% ETc in citrus can improve water productivity without causing significant yield reductions, particularly under Mediterranean or arid conditions.

**Table 1:** Irrigation requirements (IR) with different treatments.

IS & IT	Sn	ETc (mm year^− 1^)	WR (m^3^ ha^− 1^ year^− 1^)	AIR (m^3^ ha^-1^ year^-1^)
FI	1	836	8360	12865.00
	2	764	7640	11756.00
	3	802	8020	12339.00
FFDI	1	836	8360	9291.00
	2	764	7640	8491.00
	3	802	8020	8912.00
FSDI	1	836	8360	8802.00
	2	764	7640	8044.00
	3	802	8020	8443.00
DFDI	1	836	6270	6969.00
	2	764	5730	6368.00
	3	802	6015	6684.00
DSDI	1	836	6270	6602.00
	2	764	5730	6033.00
	3	802	6015	6332.00

FI: flood irrigation; DFDI: Deficit surface drip irrigation; DSDI: Deficit sub-surface drip irrigation; FFDI: full surface drip irrigation; FSDI: full sub-surface drip irrigation; IS: Irrigation System; IT: Irrigation strategies; Sn: Season; AIR: Actual applied irrigation requirements, WR: water requirements for crop or the consumptive water by crop.

### Soil, water, and plant sampling

Soil, water, and plant samples were collected at different stages of the experiment. Groundwater used for irrigation was sampled randomly under emitters across seasons. Soil samples were taken after each season at depths of 0–30 cm, 30–60 cm, 60–90 cm, and 90–120 cm using a Riverside auger. Samples were air-dried, crushed, and sieved (2 mm). Saturated soil paste was prepared and extracted after 24 h via a suction pump. Soil water content was monitoring by using PR2/6 profile prob (measures at 6 depths down to 100 cm) and HH2 datalogger as shown in (Fig. 2).

For plant samples, new growth leaves were collected before flowering each season, oven-dried at 70 °C for 24 h, milled, and packaged for analysis. Plant digestates were prepared by wet digestion of 0.5 g of dried plant material using 10 mL H_2_SO_4_ and H_2_O_2_ at 120 °C until clear, then cooled, filtered, and diluted to 50 cm³ with deionized water^34^.

### Analytical methods

The following analyses were conducted:

1. Electrical Conductivity (EC): Determined in water and soil paste extraction using a digital TDS meter (Jenway 4520, Cole-Parmer Instrument Company, USA), calibrated with standard potassium chloride (KCl) solution at 25 °C, as described by^35^.
2. pH: Measured electrometrically using a Jenway 3310 pH meter in water samples and 1:2.5 soil suspension with a calibrated combined electrode pH meter, as described by^35^.
3. Soluble Cations and Anions: Calcium (Ca²⁺), magnesium (Mg²⁺), chloride (Cl⁻), bicarbonate (HCO₃⁻), carbonate (CO₃²⁻), and sulfate (SO₄²⁻) were determined in water by titration. Sodium (Na⁺) and potassium (K⁺) were measured in water and plant digestates using a flame photometer, as described by^35^.
4. Sodium Adsorption Ratio (SAR): Calculated using the formula:

5 $$\:SAR=\:\frac{{Na}^{+}}{\sqrt{\frac{{Ca}^{2+}+{Mg}^{2+}}{2}}}$$

As described by^36^.

5. Soil Cation Exchange Capacity (CEC) and Exchangeable Sodium Percentage (ESP): Determined using the sodium and ammonium acetate method as outlined by^37^.
6. Nitrogen (N): Determined in plant digestates using the Kjeldahl method^35^.
7. Phosphorus (P): Measured in plant digestates using the Olsen method^38^.
8. Potassium (K): Measured in plant digestates by flame photometry^35^.
9. Particle Size Distribution: Determined for air-dried fine soil using the pipette method^39^.

### Initial soil properties

According to soil depth; Soil texture was ranged between heavy and light clay (International Soil Texture classification - ISSS at^40^. Soil macronutrients content was ranged between low for phosphorus (P) medium for Nitrogen (N), and high for Potassium (K)^41,42^ as shown in (Table 2). Chemical properties were derived from FI treatment as represented in (Table 2).

**Table 2:** Initial soil properties.

Depth	Particles size distribution of initial soil (%)					Available macro-nutrients (mg.kg^−1^)			Physical properties				
	C.S	F. S	Sl	Cl	Texture	*N*	*P*	K	Bd (Mg. m^− 3^)	SP (%)	FC (%)	AW (%)	PWP (%)
0–30	9.08	13.62	35.39	41.92	LCl	52.45	4.52	272.16	1.25	53.24	39.25	22.27	16.98
30–60	8.87	10.06	36	45.07	HCl	49.61	3.47	255.03	1.39	49.13	42.87	25.36	17.51
60–90	7.43	8.24	37.46	46.86	HCl	47.99	2.73	247.78	1.45	47.20	43.53	23.91	19.62
90–120	6.02	7.96	38.51	47.51	HCl	46.28	2.16	240.29	1.49	45.67	42.37	23.14	19.23

### C.S: coarse sand F.S: fine sand sl: silt cl: clay

#### LCl: light clay hcl: heavy clay N: nitrogen P: phosphorus

##### K: potassium bd: bulk density SP: saturation percent

FC: Field capacity AW: Available water PWP: Permanent wilting point.

### Irrigation water quality

The chemical characteristics of irrigation water at (Table 3), showed that the electrical conductivity ranged from 1.05 to 1.07 dS·m⁻¹, pH from 8.75 to 8.80. Sodium (Na⁺) was the dominant cation, followed by calcium (Ca²⁺), magnesium (Mg²⁺), and potassium (K⁺). Bicarbonate (HCO₃⁻) was the prime anion, followed by chloride (Cl⁻) and sulfate (SO₄²⁻). SAR between 3.43 and 3.59 across the seasons. According to^43^, the water was classified as medium saline, highly alkaline, and low sodic.

**Table 3:** Irrigation water chemical analysis.

Season	EC dSm^− 1^	pH	Soluble cations and anions mmolc. L^− 1^								SAR mmol. L^− 1^
			Na+	Ca^++^	Mg^++^	K^+^	Cl^−^	HCO_3_^−^	CO_3_^−−^	SO_4_^−−^	
1	1.07	8.78	5.56	3.00	2.00	0.16	3.50	6.00	0.00	1.22	3.52
2	1.08	8.80	5.67	4.00	1.00	0.17	3.00	6.00	0.00	1.84	3.59
3	1.05	8.75	5.42	3.00	2.00	0.11	4.00	5.00	0.00	1.53	3.43

EC: Electrical conductivity; pH: Soil reaction; Na: Sodium; Ca: Calcium; Mg: Magnesium; K: Potassium; Cl^-^: Chloride; HCO3^-^: Bicarbonates; CO3: Carbonates; SO4: Sulfates; SAR: Sodium adsorption ratio.

### Yield and water use efficiency (WUE)

Fruit yield was harvested at full maturity in January of each season and was expressed in tons per hectare (ton ha^− 1^). Water Use Efficiency (WUE) was calculated as the yield biomass produced per unit of water used, following the method described by^44^.

### Statistical analysis

Statistical analysis was performed using the Least Significant Difference (LSD) method, as described by^45^, using M Stat-C program version 2.10.

## Results

### Irrigation water, yield, and water use efficiently during three seasons

**Fig. 3:**
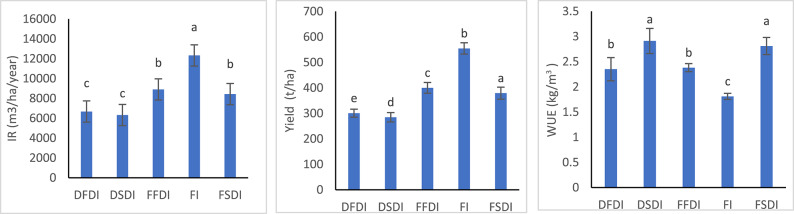
IR, Yield and WUE under different irrigation systems and strategies. FI: flood irrigation; DFDI: Deficit surface drip irrigation; DSDI: Deficit sub-surface drip irrigation; FFDI: full surface drip irrigation; FSDI: full sub-surface drip irrigation.

The data presented in Table 4; Fig. 3 show the effects of the irrigation system on yield and water use efficiency over the three growing seasons. For the FI treatment, irrigation water usage ranged from 11,756 to 12,865 m³/ha/year. The yield for this treatment was consistently high, ranging from 21.7 to 23.0 t/ha, with WUE values varying between 1.76 and 1.87 kg/m³. In contrast, the FFDI treatment, which used less water (8,491 to 9,291 m³/ha/year), resulted in lower yields (20.5 to 22.0 t/ha), but showed improved WUE, which ranged from 2.30 to 2.46 kg/m³. The FSDI treatment demonstrated both high yield (22.2 to 25.0 t/ha) and efficient water use, with WUE values from 2.63 to 2.96 kg/m³, despite using only 8,044 to 8,802 m³/ha/year of irrigation water.

The DFDI treatment, which employed a lower amount of irrigation water (6,368 to 6,969 m³/ha/year), resulted in the lowest yields among the treatments (14.0 to 17.0 t/ha), although the WUE was still relatively high, ranging from 2.09 to 2.51 kg/m³. Similarly, the DSDI treatment, which used the least amount of water (6,033 to 6,602 m³/ha/year), exhibited yield values between 16.6 and 20.0 t/ha. The WUE for DSDI ranged from 2.62 to 3.07 kg/m³, reflecting its superior water-use efficiency. Statistical analysis using the least significant difference (L.S.D. 0.05) confirmed that these differences in irrigation amounts, yield, and WUE were significant.

**Table 4:** The effect of IS and IT on IR, yield and WUE.

IS	Sn	IR (m^3^/ha/year)	Yield (t/ha)	WUE (kg/m^3^)
FI	1	12,865^a^	23.00^b^	1.79^e^
	2	11,756^a^	22.00^bc^	1.87^e^
	3	12,339^a^	21.70^bc^	1.76^e^
FFDI	1	9291^b^	22.00^bc^	2.37^d^
	2	8491^b^	20.90^cd^	2.46^d^
	3	8912^b^	20.50^cd^	2.30^d^
FSDI	1	8802^b^	25.00^a^	2.84^b^
	2	8044^b^	23.80^a^	2.96^b^
	3	8443^b^	22.20^ab^	2.63^c^
DFDI	1	6969^c^	17.00^d^	2.44^d^
	2	6368^c^	16.00^e^	2.51^d^
	3	6684^c^	14.00^e^	2.09^e^
DSDI	1	6602^c^	20.00^c^	3.03^a^
	2	6033^c^	18.50^de^	3.07^a^
	3	6332^c^	16.60^de^	2.62^c^
L.S.D (0.05)		12.62	0.05	0.06

IS: Irrigation system; FI: flood irrigation; DFDI: Deficit surface drip irrigation; DSDI: Deficit sub-surface drip irrigation; FFDI: full surface drip irrigation; FSDI: full sub-surface drip irrigation; Sn: Season; IR: Irrigation water; WUE: Water use efficiency. Within each season, means followed by the same letter in a column are not significantly different at *P* ≤ 0.05 (LSD test).

### Effect of irrigation system on soil chemical properties

**Fig. 4:**
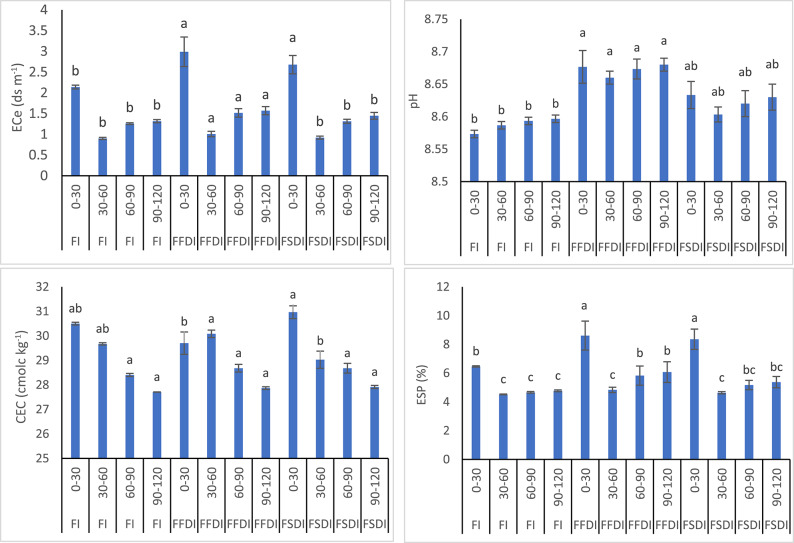
Soil chemical properties under different irrigation systems and strategies. FI: flood irrigation; DFDI: deficit surface drip irrigation; DSDI: deficit sub-surface drip irrigation; FFDI: full surface drip irrigation; FSDI: full sub-surface drip irrigation; 0–30 cm, 30–60 cm, 60–90 cm, and 90–120 cm: soil sampling depths.

The study examined the effect of various irrigation systems (FI, FFDI, and FSDI) on soil properties across three seasons, as presented in (Table 5; Fig. 4). Soil properties such as electrical conductivity (ECe, dS/m), pH, cation exchange capacity (CEC, cmolc/kg), and exchangeable sodium percentage (ESP, %) were measured at different soil depths (0–30 cm, 30–60 cm, 60–90 cm, and 90–120 cm). The results for each irrigation system and season are summarized below.

For the FI system, there was a consistent pattern observed across the three seasons, with ECe values varying from 0.87 to 2.18 dS/m at the surface (0–30 cm). These values generally decreased with depth, with the lowest ECe values observed at the 30–60 cm depth (0.87 dS/m in season 1) and the highest at the surface (2.18 dS/m in season 2). Soil pH remained relatively stable, ranging from 8.57 to 8.60 across all depths and seasons. CEC values were also stable, averaging around 29.7 to 30.5 cmolc/kg at the surface, with slight decreases with increasing depth. ESP values were highest at the surface (6.42 to 6.51%) and decreased with depth, reaching around 4.49% at the 30–60 cm depth.

For FFDI system, ECe values at the surface ranged from 2.65 to 3.36 dS/m, higher than those observed in the FI system. The surface layers (0–30 cm) exhibited the highest ECe values, with the lowest values observed at the 30–60 cm depth (0.94 to 1.07 dS/m). The soil pH for FFDI was also slightly higher than that of FI, ranging from 8.65 to 8.70 across all depths. CEC values ranged from 27.81 to 30.17 cmolc/kg at the surface, with values slightly decreasing with depth. ESP values for the FFDI system were notably higher than FI, particularly in the surface layer, reaching up to 9.66% in season 3. ESP values generally decreased with increasing depth.

In FSDI system, the ECe values ranged from 2.45 to 2.89 dS/m at the surface (0–30 cm), which were lower than those in FFDI but still higher than in FI. As with the FFDI system, the lowest ECe values were found at the 30–60 cm depth (0.88 to 0.95 dS/m). The pH values were slightly lower than those of the FFDI system, ranging from 8.59 to 8.65. CEC values for FSDI ranged from 27.85 to 31.24 cmolc/kg at the surface, with a slight decrease with depth. The ESP values for FSDI were also lower than FFDI, ranging from 6.89% at the surface to 4.55% at the 30–60 cm depth.

The statistical analysis, using the Least Significant Difference (L.S.D. 0.05), showed that there were significant differences in ECe, pH, CEC, and ESP between the different irrigation treatments and across the seasons. The highest differences were observed for ECe, with values for FFDI being significantly higher than those for FI and FSDI, particularly in the surface layers.

**Table 5:** Effect of irrigation system on soil chemical properties.

IS	Sn	Depth (cm)	ECe dSm^− 1^	pH	CEC cmolc.kg^− 1^	ESP (%)
FI	1	0–30	2.09^c^	8.57	30.49^a^	6.42^c^
		30–60	0.87	8.58	29.71	4.49
		60–90	1.25	8.59	28.33	4.66
		90–120	1.30	8.59	27.70	4.72
	2	0–30	2.18^c^	8.58	30.56^a^	6.51^c^
		30–60	0.91	8.59	29.69	4.54
		60–90	1.24	8.59	28.44	4.72
		90–120	1.29	8.60	27.71	4.84
	3	0–30	2.15^c^	8.57	30.45^a^	6.47^c^
		30–60	0.92	8.59	29.62	4.53
		60–90	1.28	8.60	28.44	4.61
		90–120	1.36	8.60	27.71	4.75
FFDI	1	0–30	2.65^b^	8.65	30.17^a^	7.65^b^
		30–60	0.94	8.65	29.95	4.64
		60–90	1.43	8.66	28.52	5.14
		90–120	1.49	8.67	27.81	5.36
	2	0–30	2.96^a^	8.68	29.68^b^	8.52^a^
		30–60	1.01	8.66	30.05	4.85
		60–90	1.49	8.67	28.69	5.87
		90–120	1.54	8.68	27.87	6.05
	3	0–30	3.36^a^	8.70	29.26^b^	9.66^a^
		30–60	1.07	8.67	30.25	5.01
		60–90	1.63	8.69	28.83	6.47
		90–120	1.68	8.69	27.92	6.80
FSDI	1	0–30	2.45^b^	8.61	30.72^a^	6.89^c^
		30–60	0.88	8.59	29.37	4.55
		60–90	1.28	8.60	28.48	4.86
		90–120	1.39	8.61	27.85	5.01
	2	0–30	2.70^b^	8.64	30.94^a^	7.73^b^
		30–60	0.93	8.61	29.04	4.66
		60–90	1.29	8.62	28.68	5.14
		90–120	1.41	8.63	27.91	5.32
	3	0–30	2.89^a^	8.65	31.24^a^	8.29^b^
		30–60	0.95	8.61	28.67	4.70
		60–90	1.37	8.64	28.88	5.51
		90–120	1.54	8.65	27.98	5.79
**L.S.D (0.05)**			**0.23**	**0.25**	**0.21**	**0.10**

IS: Irrigation system; FI: flood irrigation; DFDI: Deficit surface drip irrigation; DSDI: Deficit sub-surface drip irrigation; FFDI: full surface drip irrigation; FSDI: full sub-surface drip irrigation; Sn: Season; ECe: Electrical conductivity; pH: Soil reaction; CEC: Cation exchangeable capacity; ESP: Exchangeable sodium percentage. Significant differences (based on LSD) appear only at the 0–30 cm depth for ECe, CEC, and ESP. At deeper depths (30–60, 60–90, 90–120 cm), differences do not exceed the LSD values, so no significant statistical groupings (letters) are provided.

### Effect of irrigation strategies on soil chemical properties

**Fig. 5:**
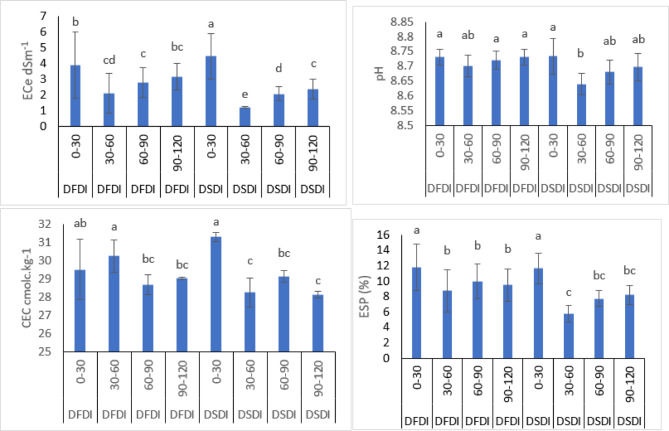
Soil chemical properties under different irrigation systems and strategies. FI: flood irrigation; DFDI: deficit surface drip irrigation; DSDI: deficit sub-surface drip irrigation; FFDI: full surface drip irrigation; FSDI: full sub-surface drip irrigation; ETo: reference evapotranspiration; ECe: electrical conductivity of the soil saturation extract; ESP: exchangeable sodium percentage; CEC: cation exchange capacity; 0–30 cm, 30–60 cm, 60–90 cm, and 90–120 cm: soil sampling depths.

**Fig. 6:**
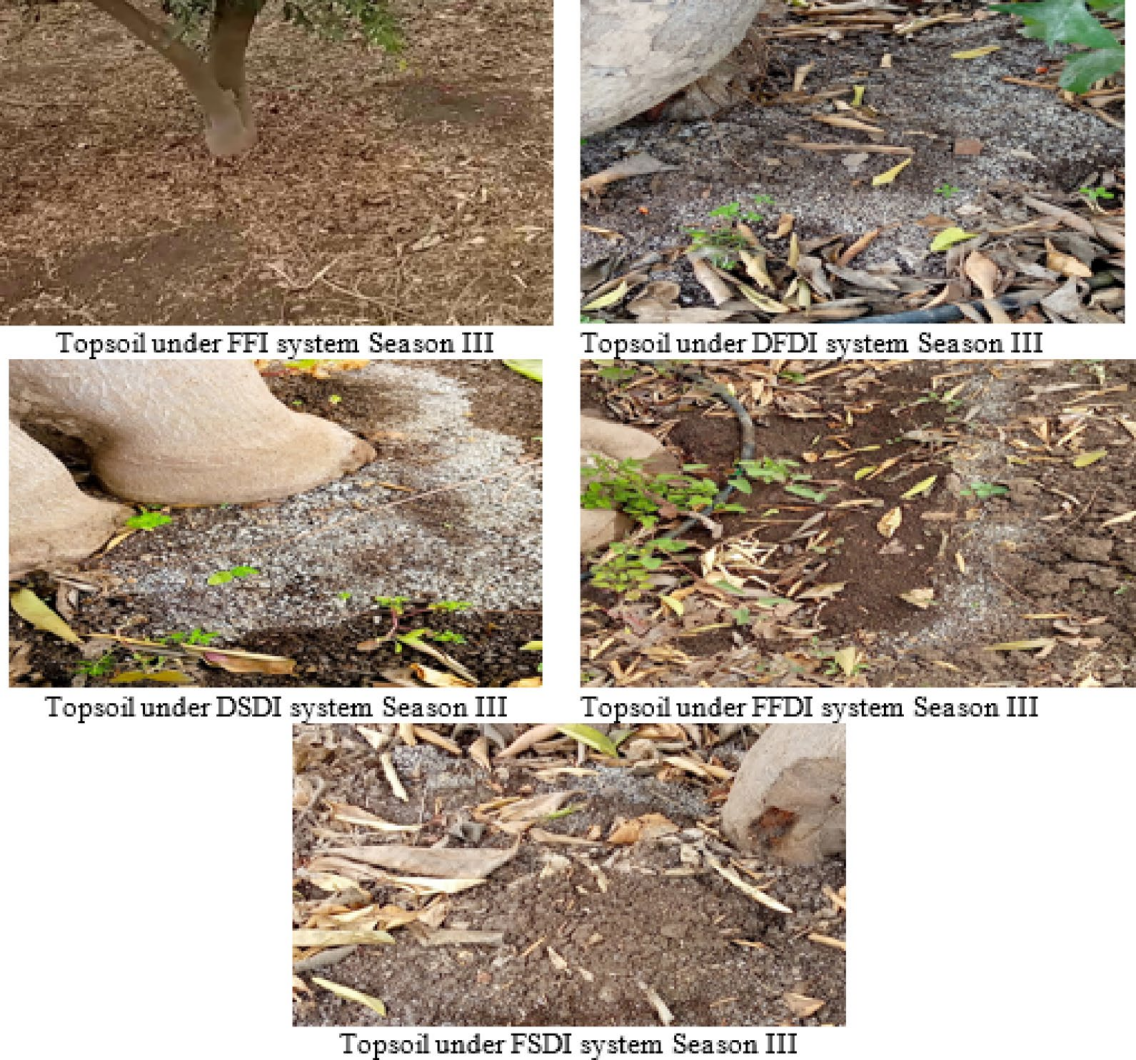
Combination effect of irrigation system and strategy on soil chemical properties. FI: flood irrigation; DFDI: deficit surface drip irrigation; DSDI: deficit sub-surface drip irrigation; FFDI: full surface drip irrigation; FSDI: full sub-surface drip irrigation.

The study evaluated the impact of two irrigation systems, DFDI and DSDI on various soil properties, as presented in (Table 6; Figs. 5 and 6). These properties include electrical conductivity (ECe, dS/m), pH, cation exchange capacity (CEC, cmolc/kg), and exchangeable sodium percentage (ESP, %), measured across three seasons and at different soil depths (0–30 cm, 30–60 cm, 60–90 cm, and 90–120 cm). The results are presented below.

For the DFDI system, ECe values were highest in the surface layer (0–30 cm), ranging from 3.98 to 8.03 dS/m across seasons. These values significantly increased from season 1 (3.98 dS/m) to season 3 (8.03 dS/m). ECe values decreased with depth, with values ranging from 1.23 to 2.75 dS/m at the 30–60 cm depth and from 2.07 to 3.94 dS/m at the 60–90 cm and 90–120 cm depths. pH values across all depths and seasons ranged from 8.67 to 8.79, showing only minor variation. CEC values were relatively stable, ranging from 27.96 to 31.28 cmolc/kg, with no substantial change across the depths, though slight decreases were noted in deeper layers. ESP values were highest in the 0–30 cm layer, with values ranging from 11.78% in season 1 to 17.58% in season 3. ESP levels decreased with depth, with values ranging from 6.55 to 12.35% at the 90–120 cm depth.

For the DSDI system, ECe values were also highest in the surface layers, ranging from 3.19 to 6.00 dS/m, and these values increased slightly over the three seasons. As with DFDI, ECe decreased with depth, with the lowest values recorded at the 30–60 cm depth (1.12 to 1.23 dS/m). The pH remained relatively stable, ranging from 8.60 to 8.79 across all depths and seasons. CEC values varied slightly between seasons, ranging from 27.38 to 31.54 cmolc/kg at the surface, with values tending to decrease in the deeper soil layers. ESP values ranged from 9.97% at the surface in season 1 to 13.88% in season 3, with lower values at greater depths, similar to the pattern observed in the DFDI treatment.

The least significant difference (L.S.D. 0.05) values were 0.19 for ECe, 0.23 for pH, 0.18 for CEC, and 0.08 for ESP, indicating that differences observed between irrigation systems and seasons were statistically significant.

**Table 6:** Effect of irrigation water strategy on soil chemical properties.

IS	Sn	Depth (cm)	ECe dSm^− 1^	pH	CEC cmolc.kg^− 1^	ESP (%)
DFDI	1	0–30	3.98^c^	8.72	29.22^c^	11.78^c^
		30–60	1.23^bc^	8.67	30.49^ab^	6.55^abc^
		60–90	2.07^bc^	8.69	28.83^c^	7.89^b^
		90–120	2.25^b^	8.70	27.96^c^	8.14^b^
	2	0–30	5.92^b^	8.76	28.00^c^	14.79^b^
		30–60	1.49^a^	8.69	30.97^a^	7.85^ab^
		60–90	2.75^ab^	8.72	29.12^bc^	9.63^a^
		90–120	3.22^a^	8.74	28.05^c^	10.37^a^
	3	0–30	8.03^a^	8.79	27.16^d^	17.58^a^
		30–60	1.72^a^	8.71	31.28^a^	8.79^a^
		60–90	3.52^a^	8.74	29.27^b^	11.82^a^
		90–120	3.94^a^	8.75	28.09^c^	12.35^a^
DSDI	1	0–30	3.19^c^	8.67	31.06^a^	9.97^c^
		30–60	1.12^c^	8.60	28.95^c^	4.89^c^
		60–90	1.65^c^	8.64	28.79^c^	6.76^bc^
		90–120	1.73^c^	8.65	27.97^c^	7.05^c^
	2	0–30	4.11^c^	8.74	31.25^a^	10.97^b^
		30–60	1.19^bc^	8.65	28.43^c^	5.34^c^
		60–90	1.99^bc^	8.68	29.18^bc^	7.64^bc^
		90–120	2.31^b^	8.70	28.14^c^	8.00^b^
	3	0–30	6.00^b^	8.79	31.54^a^	13.88^a^
		30–60	1.23^bc^	8.67	27.38^d^	6.97^b^
		60–90	2.53^ab^	8.72	29.42^b^	8.75^a^
		90–120	3.01^a^	8.74	28.34^c^	9.53^a^
**L.S.D (0.05)**			**0.19**	**0.23**	**0.18**	**0.08**

IS: Irrigation system; FI: flood irrigation; DFDI: Deficit surface drip irrigation; DSDI: Deficit sub-surface drip irrigation; FFDI: full surface drip irrigation; FSDI: full sub-surface drip irrigation; Sn: Season; ECe: Electrical conductivity; pH: Soil reaction; CEC: Cation exchangeable capacity; ESP: Exchangeable sodium percentage.

### Macro-nutrient content, sodium accumulation, and na/k ratio in soil across three seasons

**Fig. 7:**
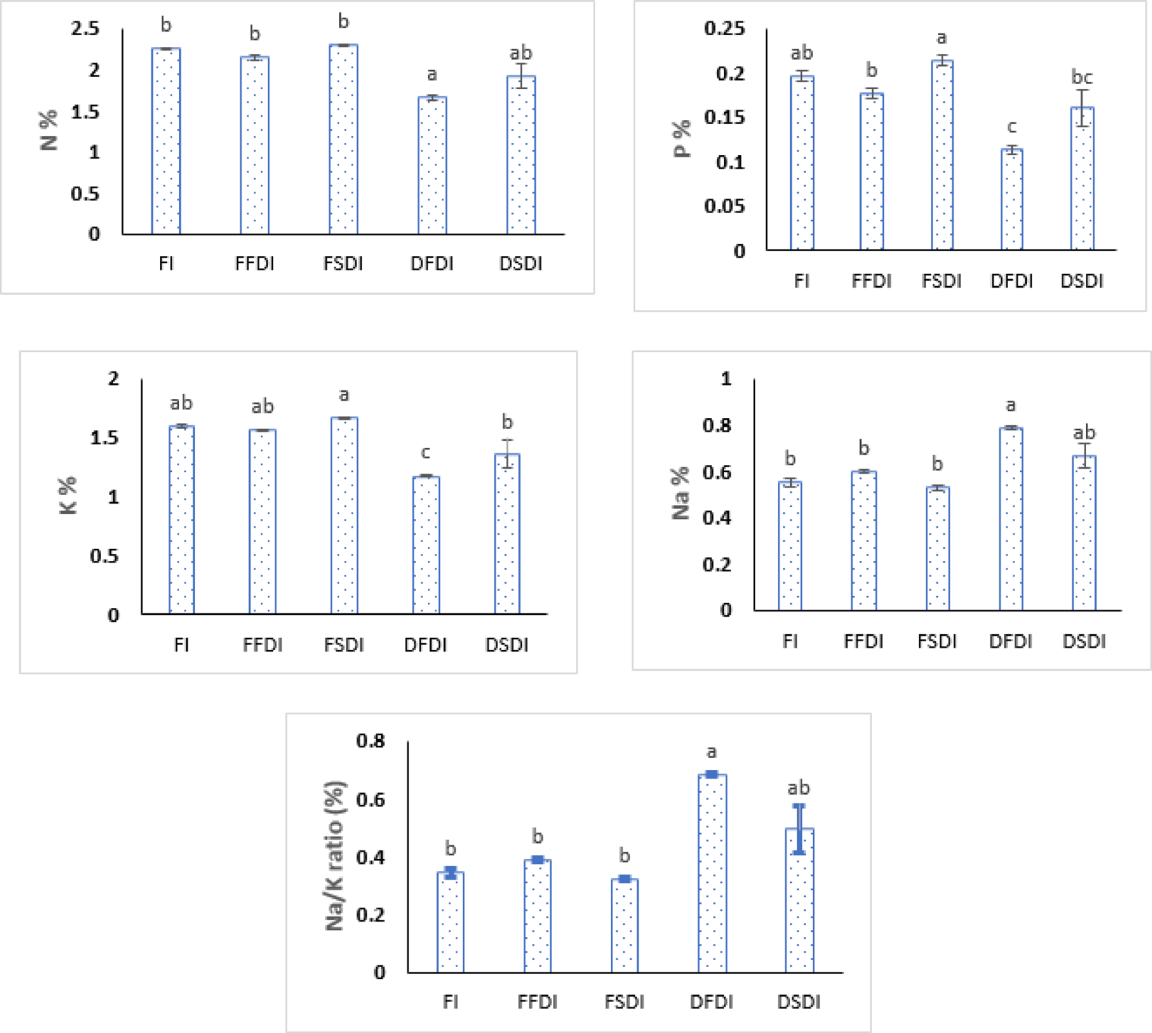
Effect of Irrigation system and water management strategy on the biological parameters of soil. N: nitrogen; P: phosphorus; K: potassium; Na: sodium; Na/K ratio: the ratio of sodium to potassium. FI: flood irrigation; DFDI: deficit surface drip irrigation; DSDI: deficit sub-surface drip irrigation; FFDI: full surface drip irrigation; FSDI: full sub-surface drip irrigation.

This study investigated the impact of different irrigation systems (FI, FFDI, FSDI, DFDI, and DSDI) on the content of macro-nutrients (N, P, K), sodium (Na), and the Na/K ratio in soil across three seasons, as presented in (Table 7; Fig. 7). The results are presented below.

For the FI system, the nitrogen (N) content ranged from 2.24 to 2.26%, phosphorus (P) content from 0.19 to 0.20%, and potassium (K) content from 1.58 to 1.61% over the three seasons. The sodium (Na) percentage varied from 0.54 to 0.57%, and the Na/K ratio ranged from 0.33 to 0.36%. There was minimal variation in macro-nutrient content and the Na/K ratio across seasons.

For the FFDI system, N content ranged from 2.11 to 2.18%, P content from 0.17 to 0.18%, and K content from 1.55 to 1.61%. Na percentage ranged from 0.59 to 0.61%, with the Na/K ratio varying between 0.38 and 0.39. As with FI, the macro-nutrient levels remained fairly stable across the seasons, though there was a slight decrease in N and P content from season 1 to season 3.

In the FSDI system, N content ranged from 2.29 to 2.31%, P content from 0.21 to 0.22%, and K content from 1.65 to 1.67%. The Na percentage was lower than in the FFDI system, ranging from 0.52 to 0.54%, and the Na/K ratio was slightly lower, ranging from 0.32 to 0.33. These results indicate relatively stable macro-nutrient levels across the three seasons, with a slight increase in N and P content over time.

For the DFDI system, N content decreased significantly across seasons, starting from 1.82% in season 1 and decreasing to 1.49% by season 3. P content also declined from 0.13 to 0.10%, while K content decreased from 1.30 to 1.01%. Na percentage, however, increased from 0.74 to 0.83%, and the Na/K ratio showed a notable increase, from 0.57 to 0.82. These trends suggest that reduced irrigation resulted in lower macro-nutrient availability but higher sodium accumulation in the soil.

In the DSDI system, N content decreased from 2.08% in season 1 to 1.80% in season 3. P content ranged from 0.14 to 0.18%, and K content decreased from 1.48 to 1.24%. The Na percentage ranged from 0.61 to 0.71%, and the Na/K ratio increased from 0.41 in season 1 to 0.57 in season 3, indicating a trend of higher sodium accumulation relative to potassium as the season progressed.

The least significant difference (L.S.D. 0.05) for N, P, K, Na, and the Na/K ratio was 0.19, 0.23, 0.18, and 0.08, respectively, indicating that the differences observed between irrigation treatments and seasons were statistically significant.

**Table 7:** Effect of irrigation system and water management strategy on the biological parameters of soil.

IS	Sn	Macro nutrients content			Na %	Na/K ratio (%)
		N %	P %	K %		
FI	1	2.26	0.20	1.59	0.54	0.34
	2	2.24	0.19	1.61	0.54	0.33
	3	2.25	0.20	1.58	0.57	0.36
FFDI	1	2.15	0.18	1.56	0.59	0.38
	2	2.11	0.18	1.56	0.61	0.39
	3	2.18	0.17	1.55	0.60	0.39
FSDI	1	2.30	0.21	1.65	0.52	0.32
	2	2.29	0.21	1.66	0.54	0.33
	3	2.31	0.22	1.67	0.53	0.32
DFDI	1	1.82	0.13	1.30	0.74	0.57
	2	1.64	0.11	1.20	0.79	0.66
	3	1.49	0.10	1.01	0.83	0.82
DSDI	1	2.08	0.18	1.48	0.61	0.41
	2	1.88	0.16	1.35	0.68	0.50
	3	1.80	0.14	1.24	0.71	0.57

N: nitrogen; P: phosphorus; K: potassium; Na: sodium; Na/K ratio: the ratio of sodium to potassium. FI: flood irrigation; DFDI: deficit surface drip irrigation; DSDI: deficit sub-surface drip irrigation; FFDI: full surface drip irrigation; FSDI: full sub-surface drip irrigation.

## Discussion

Replacing of FI by FDI system enhanced WUE under FWR strategy, while WUE was higher under FSDI than FFDI. Due to^46^ note flood irrigation reduces WUE due to evaporation and percolation losses. Surface drip (FDI) risks runoff and evaporation in clay soils, while subsurface drip (SDI) enhances WUE by targeting the root zone, reducing evaporation, and improving moisture distribution, per^47^. Meanwhile under FDI system the application of DI strategy boosts WUE temporarily by optimizing water use, as^48^ explains, with^49^ reporting 20–30% WUE gains in cotton despite lower yields, and^4^ noting higher WUE in wheat and potato under controlled deficit regimes, though with reduced yields. Increasing soil salinity by FDI application align with prior research on drip irrigation wetting patterns and soil properties affecting salt distribution^8,10,25^. The insignificant increases in soil pH across all depths under both FDI systems driven by sodium and bicarbonates in irrigation water, Soil buffering capacity and humus acids from soil organic matter decomposition^50^. Changes in CEC values reflect sodium-driven ion competition and clay dispersion^51^. Deficit irrigation saves water but impacts soil chemistry, particularly salinity, ESP, CEC, and pH. Reduced water application concentrates salts in the root zone, as^52^ noted, decreasing leaching and raising salinity, especially in high-evapotranspiration areas, per^53,54^, who found higher salinity under deficit compared to full irrigation. ESP, reflecting sodium in soil, rises with deficit irrigation, particularly with saline water, as^55,56^ observed, worsening soil structure in sodic-prone areas. CEC, tied to cation retention, may drop under deficit irrigation in low-organic-matter soils due to lower moisture, per^57^, though^58^ saw little change in clay-rich soils;^50^ linked CEC declines to sodium displacing calcium and magnesium. Soil pH increases with deficit irrigation, driven by sodium and bicarbonate buildup, as^59,60^ found, reducing leaching of basic cations and raising alkalinity, which can limit nutrient availability.

Reference^61^ found drip irrigation outperforms flood irrigation in clay soils by stabilizing water supply, enhancing nutrient uptake^61,62^. noted SDI beats SDI in clay soils by cutting evaporation and runoff, improving water and nutrient distribution^63,64^. link deficit irrigation yield drops to water stress, impairing photosynthesis and nutrient uptake via stomatal closure. Here, deficit irrigation skipped 25% leaching water, avoiding major stress, so yield losses likely stem from rootzone salinity^65^. tie salinity-induced yield drops to osmotic stress, while^66^ highlight Na⁺ and Cl⁻ toxicity^67^. note sodium outcompetes essential nutrients like K, Ca, and Mg, stunting growth^68^. tie yield losses to crop salinity tolerance.

## Conclusions

This study highlights the significant influence of irrigation systems and strategies on yield, WUE, and soil health over three citrus growing seasons. While FI achieved the highest yields (up to 23.0 t/ha) and maintained stable soil chemical properties, it required the highest water input (up to 12,865 m³/ha/year) and showed low WUE (as low as 1.76 kg/m³). In contrast, FSDI achieved the highest WUE (up to 2.96 kg/m³) and a yield of 25.0 t/ha, while reducing water use by approximately 30% compared to FI. Similarly, DSDI offered the highest WUE overall (up to 3.07 kg/m³) with the lowest water input (as low as 6,033 m³/ha/year), though with a moderate yield reduction.

FI provided the most stable soil conditions, including lower salinity and ESP values, both FFDI and FSDI treatments showed higher salinity levels in the surface layers, where the ECe at topsoil layer increased by 27.00 and 17.22% after season I under FFDI and FSDI respectively. These rates were increased to 35.77 and 23.72 after season II, then 56.46 and 34.42 after season III). ESP values have increased under both the FFDI and FSDI systems at the topsoil layer by 13.34, 17.04, and 25.42% and by 7.56, 12.58, and 17.82% for the three seasons, which may require additional soil management practices such as leaching or soil amendments to prevent salt buildup. The choice of irrigation system should therefore consider not only crop water requirements but also long-term soil health, particularly in regions prone to salinity issues. Future studies should explore strategies for mitigating the salinity and sodium accumulation in the soil under reduced irrigation systems, ensuring their sustainability and minimizing adverse effects on crop yields. Both DFDI and DSDI treatments resulted in increased soil salinity, particularly in the surface layers, as evidenced by the higher ECe and ESP values, where ECe of the topsoil layer under the DFDI system had been increased by 48.09 up to 138.72% and 36.34up to 233.71% at the subsoil layers compared with the FFDI system, while under the DSDI system, the increases of ECe had ranged between 39.18 up to 107.61% at the topsoil layer, and between 23.66 up to 177.30% at the subsoil layers compared with the FSDI system. The ESP values had been increased under DFDI and DSDI systems compared with FFDI and FSDI at topsoil by 40.86 up to 107.87% and 31.67 up to 115.46% at subsoil. These findings highlight the potential challenges of using reduced irrigation systems, such as salinization of the topsoil, which could impact long-term soil health and crop production. To mitigate these effects, it may be necessary to implement additional soil management practices, such as seasonal leaching or the use of soil amendments, to reduce sodium accumulation and prevent soil degradation. Further research could explore the long-term impact of these irrigation practices on soil health and explore methods to improve the sustainability of water use in arid regions.

FI and FSDI systems help maintain better macro-nutrient content and lower Na/K ratios. the DFDI and DSDI systems, although more water-efficient, resulted in higher sodium accumulation and nutrient imbalances. These findings underscore the importance of water management practices in ensuring soil health and nutrient availability in arid and semi-arid regions. Future studies could explore strategies to reduce sodium accumulation in reduced irrigation systems, such as incorporating soil amendments or using leaching techniques to mitigate the effects of high Na/K ratios.

## Data Availability

Data is contained within the article.

## Abbreviations

FI: flood irrigation
DFDI: Deficit surface drip irrigation
DSDI: Deficit sub-surface drip irrigation
FFDI: Full surface drip irrigation
FSDI: Full sub-surface drip irrigation
IS: Irrigation system
IT: Irrigation strategies
WUE: Water use efficiency
Sn: Season
IR: Irrigation requirements
C.S: Coarse sand
F. S: Fine sand
Sl: Silt
Cl: Clay
LCl: light clay
HCl: Heavy clay
N: Nitrogen
P: Phosphorus
K: Potassium
Na: Sodium
SAR: Sodium adsorption ratio
ESP: Sodium exchangeable capacity
CEC: Cation exchangeable capacity
EC: Electrical conductivity
pH: Soil reaction
Cl^−^: Chloride
HCO_3_^−^: Bicarbonates
CO_3_^−2^: Carbonates
SO_4_^−2^: Sulfates
ρB: Bulk density
SP: Saturation percent
FC: Field capacity
AW: Available water
PWP: Permanent wilting point
